# Implementation of Single-Fraction Lung Stereotactic Ablative Radiotherapy in a Multicenter Provincial Cancer Program During the COVID-19 Pandemic

**DOI:** 10.7759/cureus.15598

**Published:** 2021-06-11

**Authors:** Benjamin Mou, Derek Hyde, Cynthia Araujo, Leigh Bartha, Alanah Bergman, Mitchell Liu

**Affiliations:** 1 Radiation Oncology, BC Cancer Kelowna, Kelowna, CAN; 2 Medical Physics, BC Cancer Kelowna, Kelowna, CAN; 3 Radiation Therapy, BC Cancer Kelowna, Kelowna, CAN; 4 Medical Physics, BC Cancer Vancouver, Vancouver, CAN; 5 Radiation Oncology, BC Cancer Vancouver, Vancouver, CAN

**Keywords:** lung sabr, lung sbrt, single fraction, implementation, pandemic

## Abstract

Background

During the novel coronavirus disease 2019 (COVID-19) pandemic, cancer centers considered shortened courses of radiotherapy to minimize the risk of infectious exposure of patients and staff members. Amidst a pandemic, the process of implementing new treatment approaches can be particularly challenging in larger institutions with multiple treatment centers. We describe the implementation of single-fraction (SF) lung stereotactic ablative radiotherapy (SABR) in a multicenter provincial cancer program.

Materials and Methods

British Columbia, Canada has a provincial cancer program with six geographically distributed radiotherapy centers serving a population of 5.1 million, over 944,735 square kilometers. In March 2020, provincial mitigation strategies were developed in case of reduced access to radiotherapy due to the COVID-19 pandemic. SF lung SABR was identified by the provincial lung radiation oncology group as a mitigation measure supported by high-quality randomized evidence that could provide comparable outcomes and toxicity to existing fractionated SABR protocols. A working group consisting of radiation oncologists and medical physicists reviewed the medical literature and drafted consensus guidelines that were reviewed by a group of center representatives as a component of provincial lung radiotherapy mitigation strategic planning. Individual centers were encouraged to implement SF lung SABR as their resources and staffing would allow. Centers were then surveyed about barriers to implementation.

Results

On March 24, 2020, a working group was created and consensus guidelines for SF lung SABR were drafted. The final version was approved and distributed by the working group on March 26, 2020. The provincial lung radiotherapy mitigation strategy group adopted the guidelines for implementation on April 1, 2020. Implementation was completed at the first center on April 27, 2020. Barriers to implementation were identified at five of six centers. Two centers in regions with disproportionately high COVID-19 cases described inadequate staffing as a barrier to implementation. One center encountered delays due to pre-scheduled commissioning of new treatment techniques. Three centers cited competing priorities as reasons for delay. As of May 2021, two centers had active SF lung SABR programs in place, three centers were in the process of implementation, and one center had no immediate plans for implementation due to ongoing resource issues.

Conclusion

SF lung SABR was adopted by a provincial cancer program within weeks of conception through rapid communication during the development of COVID-19 pandemic mitigation strategies for radiotherapy. Although consensus guidelines were written and approved in an expedited timeframe, the completion of implementation by individual centers was variable due to differences in resource allocation and staffing among the centers. Strong organizational structures and early identification of potential barriers may improve the efficiency of implementing new treatment initiatives in large multicenter radiotherapy programs.

## Introduction

The global community experienced considerable change and challenges brought on by the novel coronavirus disease 2019 (COVID-19) pandemic. In the early days of the pandemic in March 2020, hospitals worldwide became overwhelmed by COVID-19 cases creating significant pressure on healthcare systems and infrastructure. Cancer centers delivering radiotherapy, in particular, experienced a need to consider shortened treatment regimens due to the nature of fractionated treatments delivered daily over several weeks. Minimizing hospital visits became an important strategy to not only minimize the potential for infectious exposure of patients and healthcare workers, but also to ease the strain on the healthcare system as a whole so that resources could be dedicated to the acute management of patients suffering from COVID-19. The treatment of lung cancer is especially challenging in the midst of a pandemic characterized by respiratory system compromise, as these patients are at risk from both COVID-19 as well as their underlying malignancy [1]. International experts published consensus guidelines on managing lung cancer patients with radiotherapy during the early months of the pandemic [2-6]. The recommendations from these guidelines highlight reducing treatments for early-stage non-small-cell lung cancer (NSCLC) down to a single fraction (SF) of 30-34 Gy using stereotactic ablative radiotherapy (SABR) for selected peripherally located tumors. Introducing a new approach to treatment can be challenging during a pandemic due to strained resources and competing priorities. It can be particularly difficult in larger institutions consisting of multiple geographically dispersed treatment centers. We describe the adoption and implementation of SF lung SABR in a large, multicenter, provincially coordinated cancer program.

## Materials and methods

British Columbia (BC) is the third largest province in Canada and has a population of approximately 5.1 million [7]. Residents have universal health insurance, which is delivered in five geographic health authorities. All cancer services are coordinated under a separate provincial health authority operating across the five geographically defined health authorities with clinical services administered through provincial multidisciplinary tumor groups. Radiotherapy services for the entire province are delivered by six distributed regional centers covering a surface area of approximately 944,735 square kilometers, or over twice the size of California. Following the declaration of the COVID-19 pandemic by the World Health Organization in March 2020, the provincial cancer program developed mitigation strategies in preparation for the possibility of reduced access to surgical, radiotherapy, and systemic therapy services due to the pandemic [8]. These efforts were led by the tumor group chairs and the chairs of the tumor group sub-committees. SF lung SABR was identified by the provincial lung radiation oncology sub-committee as a mitigation measure supported by high-quality randomized evidence that could provide comparable outcomes and toxicity to existing fractionated SABR protocols [9-11] and was also endorsed by international expert panels [2-6]. Lung SABR has been used in routine clinical practice in BC since 2008 and is practiced in all six centers. Until 2020, provincial guidelines recommended the use of 48 Gy in four fractions for peripheral tumors and 60 Gy in eight fractions for central tumors. As a component of the mitigation strategic planning, a working group consisting of two radiation oncologists and two medical physicists reviewed the medical literature for SF lung SABR and drafted provincial guidelines, which were then reviewed by a group of representatives from all regional centers. Communication occurred primarily by e-mail and video conference as the province was under a state of emergency limiting non-essential travel during this period. Individual centers were encouraged to implement SF lung SABR as their resources and staffing would allow given the significant disparity in new and active COVID-19 between the different health authorities across the province [12]. A virtual meeting inviting all radiation oncologists treating lung cancer in the province was held where participants were surveyed about the status of SF lung SABR at their centers as well as barriers to implementation. Descriptive data were collected and categorized based on the group discussions at the virtual meeting.

## Results

**Figure 1:**
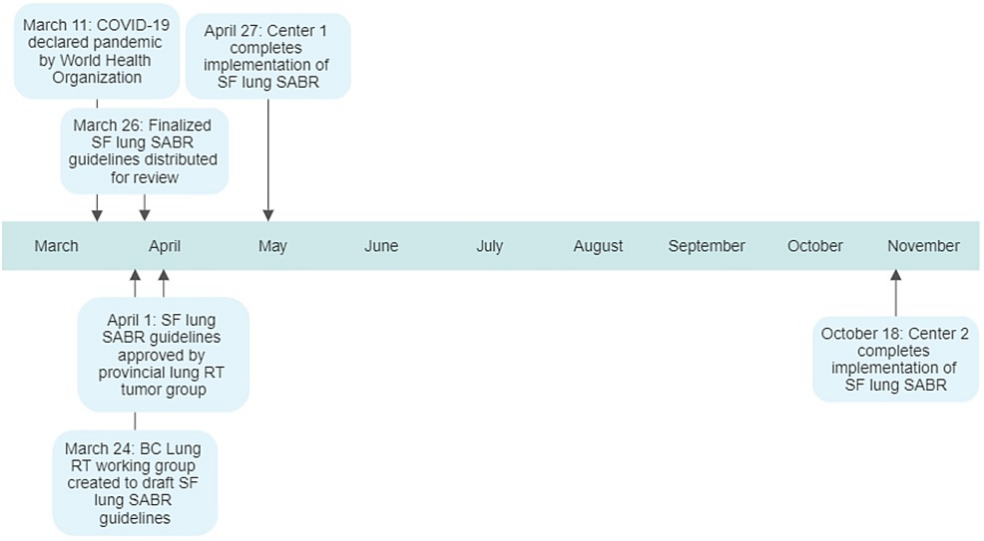
Timeline of SF lung SABR implementation in BC in 2020 SF: single fraction, SABR: stereotactic ablative radiotherapy, BC: British Columbia, RT: radiotherapy

**Figure 2:**
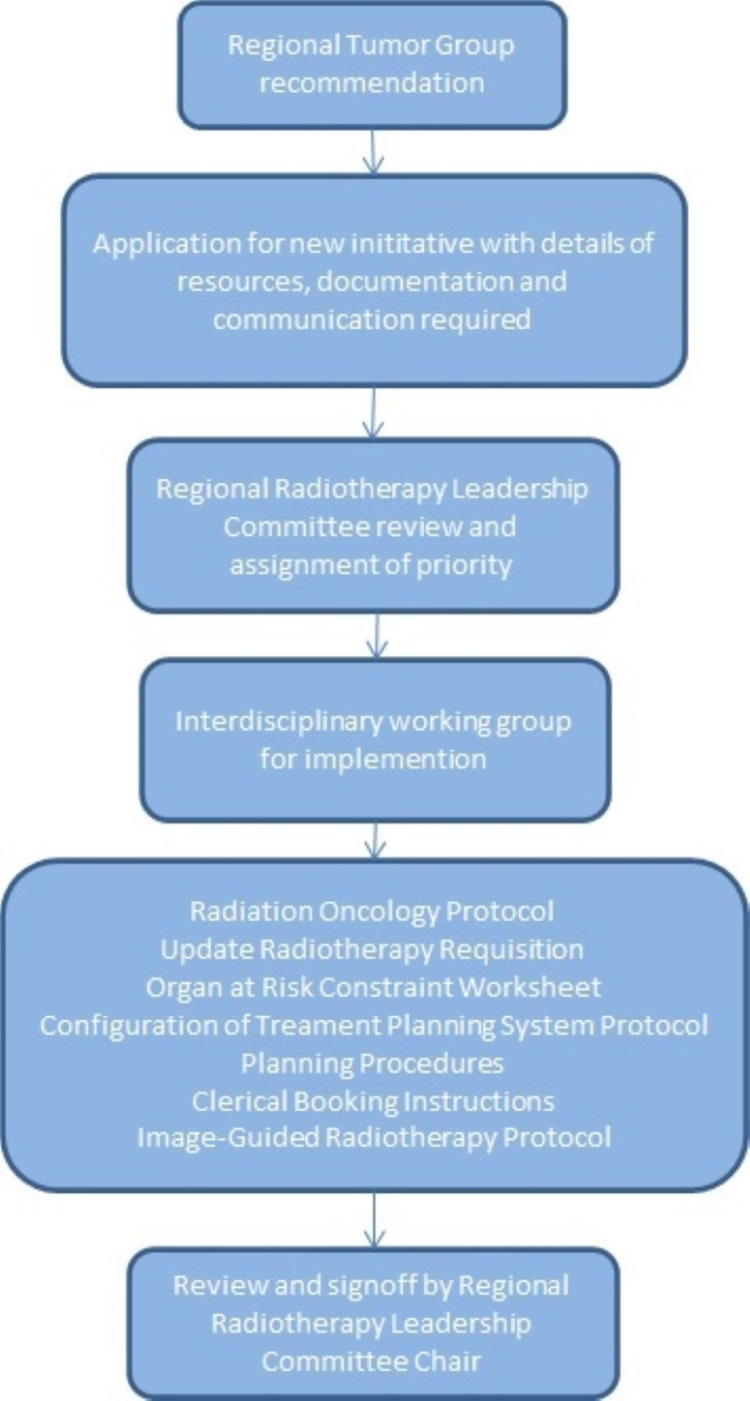
Regional workflow for new initiative implementation

A timeline of SF lung SABR implementation in BC is outlined in Figure 1. On March 24, 2020, a working group was created and consensus guidelines for SF lung SABR were drafted. The final version was approved and distributed by the working group on March 26, 2020. This addition to the provincial lung SABR guidelines identified ideal candidates for SF lung SABR as those with tumors less than 3 cm in maximum diameter, without chest wall abutment, and at least 2 cm from central thoracic organs; however, any patient with a peripheral lung tumor meeting the eligibility criteria of the Radiation Therapy Oncology Group (RTOG) 0915 trial could be considered at the discretion of the treating radiation oncologist, including tumors up to 5 cm in maximum diameter or with chest wall abutment [9]. The guidelines for SF lung SABR only applied to patients with primary early-stage NSCLC, and not patients with lung oligometastasis or oligoprogression, given the limited mature data available in the metastatic setting which were only emerging when these guidelines were written [13]. A dose of 34 Gy was suggested for tumors without chest wall abutment and 30 Gy for tumors with chest wall abutment with the goal of minimizing chest wall toxicity, while acknowledging that there may be no difference in toxicity or outcomes between either dose [14,15]. Consensus dose constraints for SF lung SABR are detailed in Table 1, which aimed to meet normal tissue tolerances with a similar biologically effective dose to those used in four-fraction lung SABR; however, strict adherence to constraints used in RTOG 0915 was acceptable [9]. The provincial lung radiotherapy mitigation strategy group adopted the guidelines for implementation on April 1, 2020. Completion of implementation, defined as activating local procedures to treat new patients, was achieved at Center 1 on April 27, 2020. Center 1 had a pre-existing, locally developed, formalized process for implementing new clinical initiatives (Figure 2). This included obtaining support from multidisciplinary leads and having written local procedures and protocols within the center prior to approval by the local radiotherapy leadership team. Center 2 adopted this workflow but encountered delays to implementation due to pre-scheduled commissioning of flattening-filter-free treatment delivery mode on existing treatment machines. Once this was finished, Center 2 completed implementation of SF lung SABR on October 19, 2020. A virtual meeting of lung radiation oncologists in the province was held on February 5, 2021. Attendees were surveyed about the status of SF lung SABR at each center and the barriers encountered. The questions and responses by center are described in Table 2. An open-ended group discussion immediately followed the survey for representatives from each center to provide additional details about their responses in order to generate discussion and provide an opportunity for attendees to share experiences to assist others. Centers 1 and 2 had fully implemented SF lung SABR with active programs in place. Centers 3, 4, 5, and 6 all cited insufficient medical physics or dosimetry support and competing center priorities as the primary barriers to implementation. Centers 3, 4, and 5 were considering implementation or in the process of implementation, while Center 6 had no immediate plans to implement SF lung SABR due to persistent resource issues. Further correspondence with each center in May 2021 demonstrated that the status of each site had not changed from the virtual meeting in February 2021. As of May 11, 2021, Centers 1 and 2 collectively treated 21 patients with SF lung SABR, resulting in a total of 63 avoided clinic visits, or approximately 32 hours of saved linear accelerator unit time.

**Table 1:** Dose constraints for single-fraction lung stereotactic ablative radiotherapy in British Columbia Dmax: Maximum dose to 0.035 cc, Vx: Volume receiving at least x Gy

Organ	Standard Constraint	Acceptable Constraint [9]
Spinal canal	Dmax ≤ 12.4 Gy	Dmax ≤ 14 Gy
Brachial plexus	Dmax ≤ 14 Gy	Dmax ≤ 17.5 Gy
	V13 Gy ≤ 3cc	V14 Gy ≤ 3cc
Proximal bronchial tree and proximal trachea	Dmax ≤ 18 Gy	Dmax ≤ 20.2 Gy
Esophagus	Dmax ≤ 15.4 Gy	Same as standard
Lungs	>1500cc ≤ 7 Gy	Same as standard
	V11 Gy ≤10%	
	Mean ≤ 4 Gy	
Heart/pericardium	Dmax ≤ 18 Gy	Dmax ≤ 22 Gy
	V15 Gy ≤ 15 cc	V16Gy ≤ 15 cc
Great vessels	Dmax ≤ 26 Gy	Dmax ≤ 37 Gy
Chest wall and ribs	Dmax ≤ 26 Gy	Dmax ≤ 30 Gy
	V18 Gy < 30 cc	V18 Gy < 30 cc
Skin	Dmax < 19 Gy	Dmax ≤ 26 Gy
	V18 Gy < 10 cc	V23 Gy ≤ 10 cc
Stomach	Dmax ≤ 12.4 Gy	Same as standard

**Table 2:** Survey questions and responses by center SF: single fraction, SABR: stereotactic ablative radiotherapy

Survey Questions	Center 1	Center 2	Center 3	Center 4	Center 5	Center 6
Is SF lung SABR available at your center?	Yes	Yes	No	No	No	No
If no, are you considering implementing SF lung SABR?	Not applicable	Not applicable	Yes	Yes	Yes	No
What are the main barriers to implementation at your center?	Not applicable	Not applicable	Lack of physics and dosimetry resources, competing priorities	Lack of physics and dosimetry resources, competing priorities	Lack of physics and dosimetry resources, competing priorities	Lack of physics and dosimetry resources, competing priorities, physician workload

## Discussion

Implementing new therapeutic initiatives in radiotherapy is typically a multidisciplinary effort requiring strong communication among colleagues in order to achieve a common goal. This can be challenging in a large institution consisting of multiple radiotherapy centers that are separated across a large geographical area, and is even more difficult in the background of a global pandemic. Established organizational structures of the provincial BC cancer program [16] provided a baseline framework for communication among physicians, physicists, and radiation therapists working in different centers across a geographically large province. The provincial lung tumor group meets regularly for weekly multidisciplinary conferences via videoconference and the provincial lung radiation oncology subcommittee typically holds annual in-person meetings in conjunction with the Canadian Lung Cancer Conference. These routine interactions among colleagues working in different centers under the same institution established pre-existing working relationships before the COVID-19 pandemic. When mitigation strategies were required in response to the pandemic, representatives from each center utilized their pre-existing relationships to rapidly communicate with one another and create a response plan in the event of significantly decreased access to radiotherapy services [8]. SF lung SABR was successfully introduced by the BC provincial cancer program within weeks of conception as a component of this provincially coordinated initiative.

Although consensus guidelines were written and approved in an expedited timeframe, the actual implementation by individual centers was variable due to differences in resource allocation and staffing among the centers. Much of the day-to-day operations of each center are administered at the local level leading to differences across centers even though they are all members of the same organization. Center 1 had a pre-existing local process for starting new initiatives, which may have streamlined the implementation process. Even though Center 1 was located in a region with a relatively high burden of COVID-19 infections, it is the largest center in terms of staff and treatment equipment, and the center has an established history of implementing new technologies in the province. Center 2 adopted the new initiative process but the main barrier for delay was the commissioning of a new treatment technology at the center. Once this was completed, Center 2 successfully completed its implementation of SF lung SABR just several months after Center 1.

**Figure 3:**
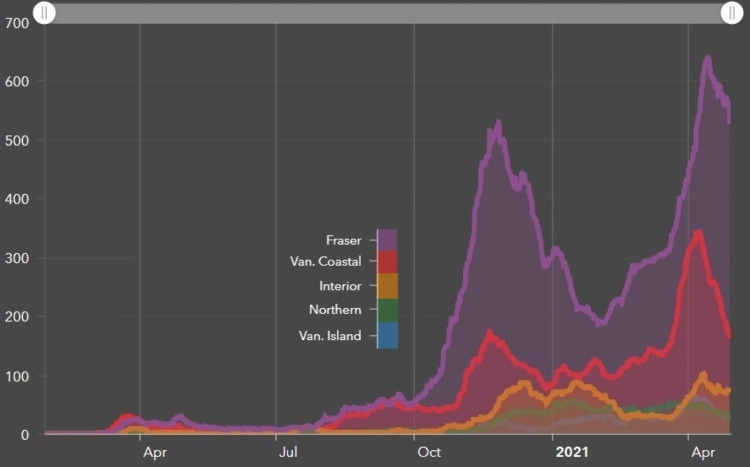
COVID-19 cases by health authority (seven-day moving average) Van: Vancouver

Not all centers implemented SF lung SABR despite interest in doing so, demonstrating that opportunities for improvement still exist. The remaining centers described inadequate staffing resources and competing priorities as the main barriers to implementation. Even though fractionated lung SABR was already in use at all centers, the implementation of SF lung SABR posed challenges about ensuring local procedures and quality assurance processes were adequate for the comfort of local multidisciplinary teams to deliver such a large dose of radiation in a single treatment. These issues may have been exacerbated by the COVID-19 pandemic as some centers were situated in regions with disproportionately high infection rates (Figure 3) [12]. COVID-19 infection rates remain high in many jurisdictions worldwide [17] and the emergence of novel coronavirus variants requires continued vigilance. Regional variation also exists across different measures of wait times, especially in lung cancer [18-20]. Depending on where a center’s pressure points along the treatment pathway lie, a center with sufficient treatment unit availability but without sufficient dosimetry or physics support may be less inclined to adopt SF lung SABR as a priority for the center, whereas centers struggling with treatment unit throughput may be more motivated to introduce SF lung SABR. Early identification of such barriers and dedicating resources to address them may improve the efficiency of implementing new treatment initiatives, particularly in large institutions with multiple treatment centers.

## Conclusions

Adoption of SF lung SABR was feasible in a large provincial cancer program consisting of multiple radiotherapy centers within weeks of conception during the COVID-19 pandemic. Rapid communication through efficient organizational structures aided this endeavor; however, variations in the completion of implementation in some centers highlighted the differences in resource allocation and COVID-19 infection rates across the province. Early identification of potential barriers and challenges may improve the efficiency of implementing new treatment initiatives in large multicenter radiotherapy programs. SF lung SABR among other appropriate shortened radiotherapy regimens may help mitigate the risk of COVID-19 to patients and treatment staff by limiting clinic visits, and as a result may improve efficiency in the system as a whole, so resources can then be redirected to other areas of need.
